# Bolingbroke Hospital

**Published:** 1904-05-28

**Authors:** M. Mackintosh

**Affiliations:** Hon. Sec. Frankfort House, Clapham Common, S.W.


### EDITOR'S LETTER-BOX.

Our Correspondents are reminded that prolixity is a great bar to publication, and that brevity of style and conciseness of statement greatly facilitate early insertion.]

BOLINGBROKE HOSPITAL.

*To the Editor of* THE HOSPITAL.

SIR,—I am instructed by the Council of the South-West London Medical Society to forward you the following resolution passed by them to-day: "That having seen the adverse opinion recently passed on Bolingbroke Hospital, the Council of the Society would like to take this opportunity of expressing their appreciation of the attention and treatment the patients receive from the medical officers and matron."

I am, Sir, yours obediently,

M. MACKINTOSH, Hon. Sec.

Frankfort House, Clapham Common, S.W.,

May 18, 1904.

